# The impact of skeletal muscle mass on survival outcome in biliary tract cancer patients

**DOI:** 10.1371/journal.pone.0204985

**Published:** 2018-10-10

**Authors:** Panita Limpawattana, Daris Theerakulpisut, Kosin Wirasorn, Aumkhae Sookprasert, Narong Khuntikeo, Jarin Chindaprasirt

**Affiliations:** 1 Division of Geriatric Medicine, Department of Internal Medicine, Faculty of Medicine, Khon Kaen University, Khon Kaen, Thailand; 2 Division of Nuclear Medicine, Department of Radiology, Faculty of Medicine, Khon Kaen University, Khon Kaen, Thailand; 3 Medical Oncology Unit, Department of Internal Medicine, Faculty of Medicine, Khon Kaen University, Khon Kaen, Thailand; 4 Division of Hepatobiliary Surgery, Department of Surgery, Faculty of Medicine, Khon Kaen University, Khon Kaen, Thailand; University of Nebraska Medical Center, UNITED STATES

## Abstract

Low skeletal muscle mass is frequently observed in cancer patients and is known to be a poor prognostic factor for survival outcomes. The purposes of our study were to determine the prevalence of sarcopenia and its relation to mortality in biliary tract cancer. Body composition measurements (skeletal muscle index, total fat mass, bone mineral content) were evaluated by using dual-energy x-ray absorptiometry in 75 biliary tract cancer patients before chemotherapy. Muscle strength was measured by handgrip strength and gait speed. Overall survival and its associated factors were determined. The mean appendicular muscle mass was 17.8±2.7 kg in men and 14.0±2.1 kg in women (p < 0.05). Sarcopenia was diagnosed in 46 patients (61.3%) and higher proportion of men was classified as sarcopenia than women (69.0% vs 35.3%, p < 0.05). Multivariable analysis adjusted for chemotherapy regimen and age revealed that high appendicular muscle mass independently predicted better survival outcomes (HR 0.40; 95% CI, 0.18 to 0.88; p = 0.023). Sarcopenia is common in biliary tract cancer patients and low appendicular muscle mass was associated with poor survival outcome.

## Introduction

Biliary tract cancer is common in Thailand, but the prevalence is increasing worldwide [1, 2]. Even though there is a screening program, most of the tumors detected are in advanced stage [3]. Most BTC patients are not eligible for curative resection resulting in a dismal prognosis, with a median survival of 8–12 months despite palliative chemotherapy [4]. The pooled analysis of two randomized phase III trials showed the superior survival with cisplatin-gemcitabine group with a median survival of 11.6 months compared with 8.0 months in gemcitabine alone group [5]. However, the response rate was only 19% in the bile duct tumor subgroup and there were nearly 70% of patients suffered from grade 3 or 4 adverse events [6]. Unquestionably, we have restricted access to predict who will get the largest survival benefit prior to the treatment.

Although tumor biology is a vital part determining the treatment outcome, patient factors also play a major role. Even though ECOG performance status is widely used to assess the suitability for combination chemotherapy, it is subjective to assessors and there was a high inter-observer variability [7, 8].

Low muscle mass and poor physical function or sarcopenia is classified according to definitions based on the appendicular muscle mass, which is measured by dual x-ray absorptiometry (DXA) scan [9]. Wasting of skeletal muscle has been shown to be associated with increased chemotherapy and targeted drugs adverse events, postoperative complications, and as a predictor of poor survival [10–13]. These findings suggest that low muscle mass may be an objective measure of the tolerability of treatment and should be considered in treatment planning and decision-making regarding fitness for combination chemotherapy.

Accordingly, the objective of the current study was to describe the prevalence of sarcopenia among BTC patients and to investigate its impact on mortality.

## Materials and methods

This single-center, prospective study included patients with BTC undergoing first-line chemotherapy with fluoropyrimidine or gemcitabine-based regimen from January 2016 to September 2017 in Srinagarind Hospital, Khon Kaen University. Inclusion criteria were age of at least 18 years old, histological or cytological proven biliary tract cancer, ECOG performance status of 0–1, and adequate organ function. Patients were excluded if they had a second malignancy, had other active medical illnesses, were unable to undergo the dual-energy X-ray absorptiometry (DXA), or had conditions that would affect the DXA results. Gemcitabine was administered weekly with either cisplatin or carboplatin as physician’s choice (8 cycles of 1000 mg/m^2^ day 1, 8 per 3 weeks) i.e., a total of 24 weeks. Another regimen was platinum combined with 5-fluorouracil 1000 mg/m^2^ every 3 weeks i.e., a total of 24 weeks. This study was approved by the Khon Kaen University Ethics Committee as instituted by the Declaration of Helsinki (Number HE581333). Written informed consents were obtained from all patients.

### Body composition and function measurements

Body composition measurement was performed before chemotherapy and consisted of the measurement of muscle, fat, and bone mass by Dual-energy x-ray absorptiometry (DXA). Appendicular muscle is the sum of muscle mass of upper and lower extremities. Appendicular muscle corrected for height, resulting in an appendicular muscle index (ASMI) in kg/m^2^. Physical performance was evaluated by the 6-min walk distance. Handgrip strength was measured by a handheld dynamometer (GRIP-D (T.K.K.5401) model). Patient was in an upright position and held the dynamometer in a dominant hand with the instrument held down at the side of the body and the arm is fully extended.

Sarcopenia diagnosis is based on the criteria of the Asian Working Groups of sarcopenia (AWGs) consisting of low muscle mass and low muscle strength. The cutoff values for sarcopenia were; ASMI of ≤7.0 kg/m^2^ for men and ≤5.4 kg/m^2^ for women, and either a gait speed of <0.8 m/s or handgrip strength of <26 kg for men and <18 kg for women [9].

### Statistical analysis

Baseline clinical characteristics were analyzed using descriptive statistics. Percentages were used to describe categorical variables. Continuous variables were described as mean ± standard deviation (SD) or as median ± interquartile range (IQR) if the data was not normally distributed. Simple and multiple linear regression analyses were performed to identify factors contributing to appendicular muscle. The independent variables with a p-value <0.20 or clinical significance in literature review were then entered a multiple linear regression model. Multicollinearity was checked by calculating variance inflation factors.

Overall survival (OS) was defined as the time from the date of diagnosis until the date of death or the end of follow-up (31^st^ March 2018). Survival analysis was performed using Kaplan-Meier method and log-rank test. Univariable and multivariable Cox-proportional hazard model were performed to determine the factors associated with survival. A p-value of < 0.05 was considered to be statistically significant in all tests. All data analyses were carried out using STATA software (StataCorp LP, College Station, TX, USA).

## Results

A total number of 75 biliary tract cancer patients were included, of which 35 were intrahepatic tumor and 38 were either perihilar or distal type. The median age was 57 years (range, 43–77 years); 58 patients (77.3%) were men (Table 1). In total, 20 patients (27%) received less than half of planned chemotherapy, resulting in a median of 4 cycles of chemotherapy in the entire cohort. Sixty patients (80%) were treated with platinum/5-fluorouracil while 15 patients (20%) underwent treatment with platinum/gemcitabine.

**Table 1:** Baseline clinical characteristics, values expressed as n (%).

Characteristics	n = 75
Age (years)	
Median (IQR)	57
Range	43–77
Male sex	58 (77.3)
ECOG-PS	
0	40 (53.3)
1	35 (46.7)
Location of primary tumor	
Intrahepatic	35 (47.9)
Extrahepatic	38 (52.1)
TNM stage II/III/IV	6/5/64
Node-positive	50 (66.7)
Metastasis	45 (60)
Liver surgery	26 (34.7)
Biliary drainage	15 (20)
Chemotherapy regimen	
Fluorouracil-based	60 (80)
Gemcitabine-based	15 (20)

### Body composition and physical function

The median BMI was 21.3 kg/m^2^ (IQR: 18.9–23.7) in men and 21.8 kg/m^2^ (IQR: 19.1–25.2) in women (Table 2). Mean fat mass was significantly higher in women (p < 0.05). There was a statistically significant difference in mean appendicular muscle between genders; the mean appendicular muscle in men was 17.8±2.7 kg and 14.0±2.1 kg in women (p < 0.05). Sarcopenia was diagnosed in 46 patients (61.3%) and higher proportion of men than women were classified as sarcopenia (69.0% vs 35.3%, p < 0.05). Regarding the physical function, the median handgrip strength was 17.9 kg in men and 15.6 kg in women without statistical significance (p = 0.14). The gait speed was indifferent between both sexes (p = 0.67).

**Table 2:** Body composition and function, values given as median (IQR), unless state otherwise.

Characteristic	Males (n = 58)	Females (n = 17)	Total (n = 75)
Body weight (kg)	57.5 (50.5, 61.8)	54.7 (48, 62.4)	56.7 (49.2, 62)
Height (cm)	165 (160, 167)	155 (155, 159)	163 (158, 166)
BMI (kg/m^2^)	21.3 (18.9, 23.7)	21.8 (19.1, 25.2)	21.5 (19, 24)
Underweight (≤ 18.5 kg/m^2^), n (%)	12 (20.7)	2 (11.8)	14 (18.7)
Normal (18.5–24.9 kg/m^2^), n (%)	37 (63.8)	10 (58.8)	47 (62.7)
Overweight (25–29.9 kg/m^2^), n (%)	7 (12.1)	5 (29.4)	12 (16.0)
Obesity (≥30.0 kg/m^2^), n (%)	2 (3.5)	0	2 (2.7)
Weight loss (kg)	6.4 (2, 11.2)	3.3 (1, 6.7)	5 (2, 9.8)
Skeletal mass (kg), mean (SD)			
Upper extremities	4.8 (0.8)	3.3 (0.5)	4.5 (0.9)
Lower extremities	13.0 (1.9)	10.7 (1.7)	12.5 (2.1)
Appendicular muscle mass	17.8 (2.7)	14.0 (2.1)	16.9 (3.0)
ASMI (kg/m^2^), mean (SD)	6.6 (0.9)	5.8 (0.8)	6.4 (1.0)
Bone mineral content (kg), mean (SD)	2.6 (0.4)	2.1 (0.4)	2.4 (0.4)
Fat (kg), mean (SD)	12.0 (6.9)	16.8 (6.0)	13.1 (7.0)
Handgrip strength (kg)	17.9 (14.1, 24.2)	15.6 (12.9, 18.5)	17.2 (14, 21.2)
Gait speed (m/s)	0.08 (0, 0.18)	0.06 (0, 0.16)	0.08 (0, 0.17)
Sarcopenia, n (%)	40 (69)	6 (35.3)	46 (61.3)
Osteoporosis, n (%)	10 (17.2)	1 (5.9)	11 (14.7)

**Abbreviation**: BMI: body mass index, ASMI: Appendicular skeletal muscle index.

### Association between clinical factors and appendicular muscle mass

**Fig 1:**
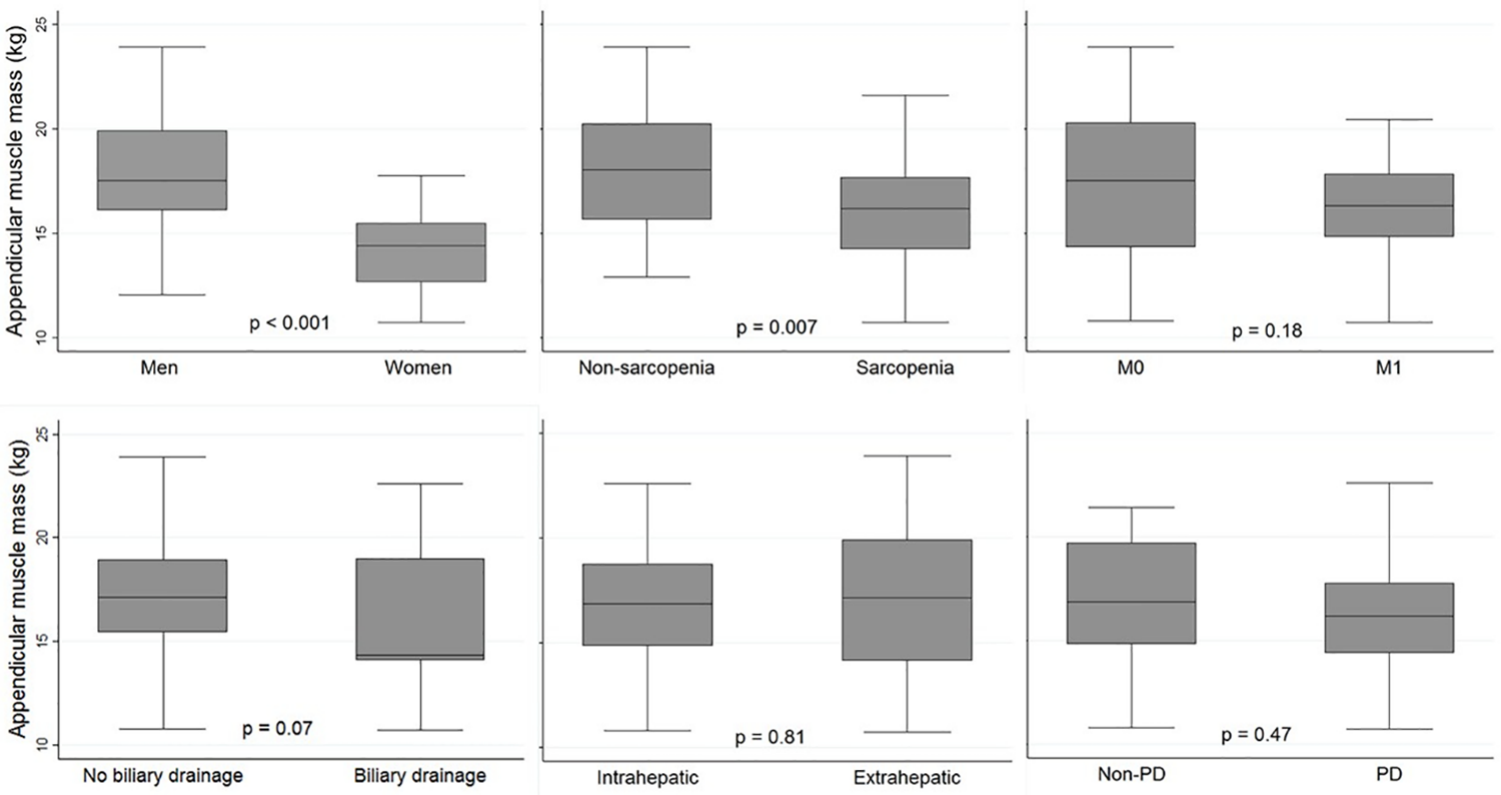
Appendicular muscle mass in each subset was shown with a p-value of Wilcoxon rank-sum test. The median was presented as the black line. The top and bottom of each box indicated the upper and lower quartiles of the samples and the bars represented the minimum and maximum values. M, metastasis; PD, progressive disease.

Low appendicular muscle mass was observed in female gender, sarcopenia, patients with metastasis, and biliary drainage (Fig 1). Female patients had significantly lower appendicular mass than male patients (p<0.001). Patients with sarcopenia also had lower muscle mass (p < 0.05). There was no statistical association between primary tumor location or response to chemotherapy and appendicular muscle.

**Fig 2:**
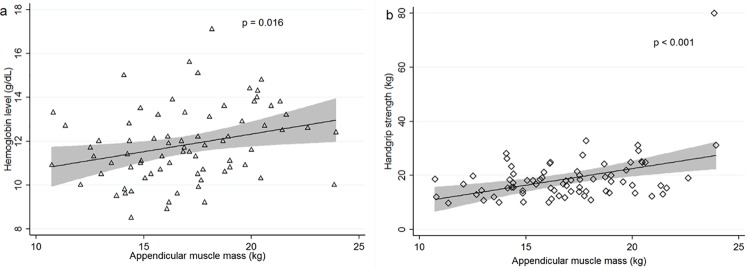
Association between appendicular muscle mass and hemoglobin level (a) and handgrip strength (b). The grey area indicates the 95% confidence interval.

There was a positive linear association between hemoglobin level and appendicular muscle mass (β = 0.48, p = 0.016, Fig 2a). And there was also a statistically significant linear association between appendicular muscle mass and handgrip strength (β = 0.13, p < 0.001, Fig 2b). Advanced age was associated with decreased appendicular mass; for each one-year increase in age, there is a decrease in muscle mass of 110 g. A multiple regression was performed to predict appendicular muscle mass from gender, age, BMI, hemoglobin level, and handgrip strength. Only three variables (age, female gender, and BMI) significantly predicted appendicular muscle mass, with R^2^ = 0.569 (Table 3). No severe collinearity between variables was detected with variation inflation factors between 1.1 and 1.3.

**Table 3:** Simple and multiple linear regression analysis using appendicular muscle mass as the dependent variable.

	Simple linear regression			Multiple linear regression		
	Coefficient	95% CI	p-value	Coefficient	95% CI	p-value
Age (years)	-0.11	-0.19, -0.04	0.004*	-0.10	-0.16, -0.04	0.002*
Female	-3.71	-5.11, -2.31	<0.001*	-3.96	-5.15. -2.76	<0.001*
BMI (kg/m^2^)	0.28	0.09, 0.47	0.005*	0.23	0.09, 0.38	0.002*
Hb (g/dL)	0.48	0.09, 0.87	0.016*	0.17	-0.12, 0.46	0.252
Albumin (g/dL)	0.75	-0.54, 2.05	0.250	-		
Handgrip strength (kg)	0.13	0.06, 0.20	<0.001*	0.05	-0.01, 0.11	0.077
Biliary drainage	-1.50	-3.19, 0.19	0.081	-		

Dependent variable, appendicular muscle mass; Adjusted R^2^ = 0.569; the level of significance at p < 0.05; CI, confidence interval; BMI, body mass index; Hb, hemoglobin

### Effect of appendicular muscle mass on survival

**Fig 3:**
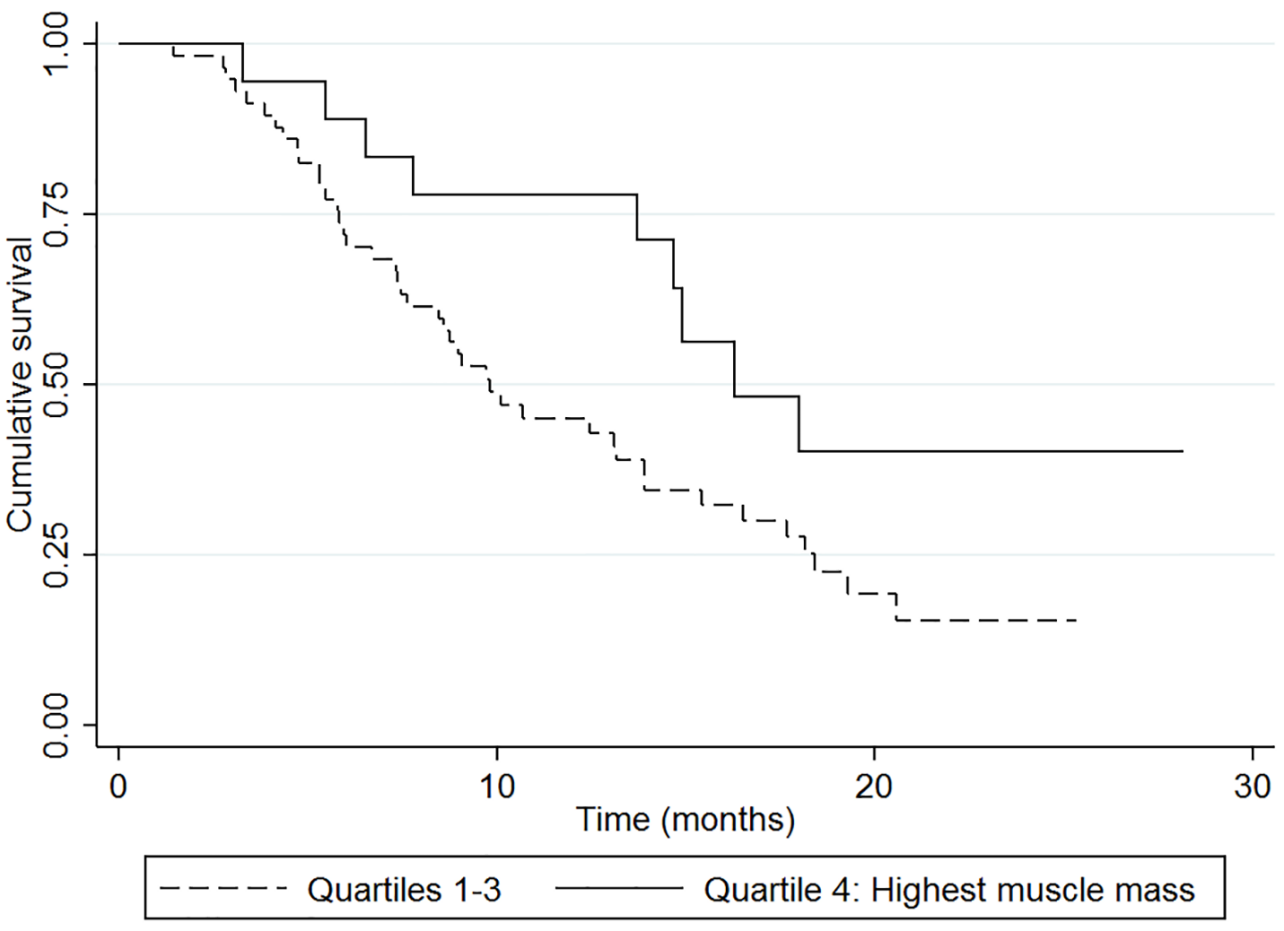
Patients with appendicular muscle mass of ≥ 19.0 kg (quartile four, highest amount of muscle mass) had a significantly longer overall survival compared with those with low appendicular muscle (quartiles one to three); HR 0.46, 95% CI 0.22–0.95, p = 0.037.

At the follow-up time, 52 patients (69.3%) had died. The median survival of the entire cohort was 13.2 months (95% CI 8.8–15.4 months). Patients with appendicular muscle mass of 19.0 kg or more (quartile four; highest muscle mass) had significantly longer overall survival compared with those with low muscle mass (quartiles 1–3) with a hazard ratio (HR) of 0.46 (95% CI 0.22–0.95, p = 0.037) (Fig 3).

BMI was not significantly associated with overall survival; however, obese patients were at highest risk for poor survival outcome. The median survival for underweight, normal-weight, overweight, and obese patients were 10.7, 13.9, 9.7, and 3.3 months respectively (p = 0.06).

In the final survival model, two independent variables including neutrophil-lymphocyte ratio (NLR) and high appendicular muscle mass were significant predictors of OS (Table 4). The adjusted hazard ratios (95% confidence interval) of these two factors were 1.17 (1.01, 1.35) and 0.40 (0.18, 0.88), respectively. There was no significant violation of the proportional hazards assumption.

**Table 4:** Univariable and multivariable Cox proportional hazards models for overall survival.

	Univariable			Multivariable		
	HR	95% CI	p-value	HR	95% CI	p-value
Appendicular muscle						
Quartile 1–3	Ref	Ref	Ref	Ref	Ref	Ref
Quartile 4	0.46	0.22, 0.95	0.037*	0.40	0.18, 0.88	0.023
NLR	1.26	1.11, 1.42	<0.001	1.17	1.01, 1.35	0.039
Chemotherapy						
Carboplatin-based	Ref	Ref	Ref	Ref	Ref	Ref
Cisplatin-based	0.50	0.28, 0.89	0.021	0.69	0.35, 1.36	0.280
Age (years)	0.99	0.96, 1.03	0.607	0.97	0.94, 1.01	0.185

HR, Hazard ratio; CI, confidence interval; NLR, neutrophil-lymphocyte ratio

## Discussion

To our knowledge, this is one of the first studies exploring the relationship between body composition and survival in biliary tract cancer. We noted several findings of interest. First, in this cohort, sarcopenia at diagnosis was 61.3% and skeletal muscle mass depletion at diagnosis predicted worse survival for advanced biliary tract cancer patients.

Sarcopenia was observed in more than half of the patients (69% of male patients and 35.3% of female patients; Table 2). It was comparable to previous reports for advanced stage cancers including esophageal, pancreatic, renal cell and urothelial carcinoma [13–16]. However, it is much higher than those reported in colorectal cancer [17, 18]. Tumor biology and natural history of disease differed. Colon cancer, even in advanced stage, the prognosis is fair with multiple options of treatment including multi-chemotherapy and targeted therapy.

We showed that low appendicular muscle mass was independently associated with increased all-cause mortality. Patients with BTC with the highest muscle mass (highest quartile) were at a significantly increased survival on multivariable analysis (HR 0.40; 95% CI, 0.18 to 0.88). The survival curve split early in the course, thereby highlighting the value of muscle mass as a baseline prognostic factor before chemotherapy. The results are in line with those reported in various malignancies, where low muscle mass was independently associated with reduced survival [11, 14, 19].

The hypotheses of sarcopenia as a risk of poor survival include inflammation, low immune activity, and inactivity. However, the mechanisms remain uncertain why it increases the risk of mortality. Neutrophil-lymphocyte ratio (NLR) was also the independent predictor for survival outcomes. There is increasing evidence indicates that inflammatory marker, particularly NLR, is a useful biomarker for recurrence and all-cause mortality [20, 21]. The meta-analysis of a total of 2093 patients with cholangiocarcinoma indicated that patients with high NLR had unfavorable OS outcomes [22]. Nevertheless, we did not find association between NLR and muscle depletion or sarcopenia in this cohort.

Aging is the most important physiological factor for muscle wasting leading to poor physical performance and frailty. In our cohort, we found that advanced age was correlated with decreased lean muscle mass, for each 1-year age increase, appendicular muscle mass decreased 0.10 kg. Since the incidence of elderly cancer is increasing [23, 24], evaluation of muscle mass along with geriatric assessment in elderly patients receiving chemotherapy is warranted. Female gender was also a risk for decreased muscle mass. Even though female patients had greater BMI compared to male patients, the muscle mass was significantly lower, but the fat mass was higher. Both ageing and gender were important factors determining the muscle mass but both factors were not associated with survival. In this cohort, the muscle mass was evaluated before the start of chemotherapy. The drug thus could not affect the loss of mass.

In this study, we assessed hemoglobin level before chemotherapy and found that it was positively correlated with appendicular muscle mass. The results were consistent with those reported from a study of 909 participants 65 years and older, which found that hemoglobin levels were significantly associated with muscle density and muscle area (measured by computed tomography) [25]. This may have been related to the nutritional status, coexistent of anemia of chronic disease, and frailty. Similarly, the functional assessment of grip strength was also associated with appendicular muscle mass. Because skeletal muscle has been linked to nutritional status, the plasma albumin level was quantified before chemotherapy, but we did not find any significant differences between the low- and high-muscle groups. A meta-analysis which included 2125 individuals demonstrated that even in the context which malnutrition was obviously observed, the serum albumin levels were normal[26]. Hence, the role of albumin as a measure of nutritional status might be weak but could be used as a complement to other clinical factors.

BMI was significantly associated with skeletal muscle mass and there was a trend toward survival outcome. Survival was better in the normal-weight group, while obese patients had the shortest overall survival. The findings were consistent with previous studies of both biliary tract cancer and other cancer types [27–28]. The obesity paradox pattern of survival that was mentioned by Lennon et al [29] was also demonstrated.

The strength of this study was that muscle mass was measured by DXA scan which evaluated the whole skeletal muscle and data regarding muscle function was assessed prospectively. We defined sarcopenia according to the international consensus definitions by AWGS, which are sex- and race- specific for Asian people and include both the muscle mass and muscle strength; the handgrip strength and overall performance by measuring gait speed [9]. However, it is noteworthy to mention that the lumbar skeletal muscle index at L3 from computed tomography is still the widely used method to measure sarcopenia and has been validated in various cancers [11, 15, 19]. It is a convenient and could be obtained simultaneously with the imaging for biliary tract cancer.

Interestingly, the rate of sarcopenia was high even though we included only patients with ECOG performance status of 0 or 1. This leads us to conclude that the general health conditions in cholangiocarcinoma patients are poor and sarcopenia could be occult.

The main limitation is the relatively small sample size, particularly in subgroup analysis. The cross-sectional design of our study cannot explain the change of muscle mass after chemotherapy. Further longitudinal studies are warranted to evaluate the chemotherapy effect to muscle mass and its effect to survival.

## Conclusion

Sarcopenia was common in BTC even in good performance status patients. Low muscle mass was associated with advanced age, female gender, and low BMI and it was an independent prognostic factor for survival. Since there is no molecular biomarker that predict outcome of chemotherapy, body composition should be considered as a potential marker. As such, we propose that interventions to modify skeletal muscle wasting may result in better outcome before and during chemotherapy.

## Supporting information

S1 Table Clinical characteristics of 75 biliary tract cancer patients. (XLS)
